# WT1 Trio Peptide-Based Cancer Vaccine for Rare Cancers Expressing Shared Target WT1

**DOI:** 10.3390/cancers15020393

**Published:** 2023-01-06

**Authors:** Yusuke Oji, Naoki Kagawa, Hideyuki Arita, Norifumi Naka, Ken-ichiro Hamada, Hidetatsu Outani, Yasushi Shintani, Yoshito Takeda, Eiichi Morii, Kenzo Shimazu, Motoyuki Suzuki, Sumiyuki Nishida, Jun Nakata, Akihiro Tsuboi, Miki Iwai, Sae Hayashi, Rin Imanishi, Sayaka Ikejima, Mizuki Kanegae, Masahiro Iwamoto, Mayu Ikeda, Kento Yagi, Haruka Shimokado, Hiroko Nakajima, Kana Hasegawa, Soyoko Morimoto, Fumihiro Fujiki, Akira Nagahara, Atsushi Tanemura, Yutaka Ueda, Tsunekazu Mizushima, Masato Ohmi, Takayuki Ishida, Manabu Fujimoto, Norio Nonomura, Tadashi Kimura, Hidenori Inohara, Seiji Okada, Haruhiko Kishima, Naoki Hosen, Atsushi Kumanogoh, Yoshihiro Oka, Haruo Sugiyama

**Affiliations:** 1 Department of Clinical Laboratory and Biomedical Sciences, Osaka University Graduate School of Medicine, Osaka 565-0871, Japan; 2 Department of Neurosurgery, Osaka University Graduate School of Medicine, Osaka 565-0871, Japan; 3 Department of Neurosurgery, Osaka International Cancer Institute, Osaka 541-8567, Japan; 4 Department of Orthopedic Surgery, Nachikatsuura Town Onsen Hospital, Nachikatsuura, Wakayama 649-5331, Japan; 5 Hamada Orthopedic Surgery, Kawanishi City 666-0021, Japan; 6 Department of Orthopedic Surgery, Osaka University Graduate School of Medicine, Osaka 565-0871, Japan; 7 Department of Thoracic Surgery, Osaka University Graduate School of Medicine, Osaka 565-0871, Japan; 8 Department of Respiratory Medicine and Clinical Immunology, Osaka University Graduate School of Medicine, Osaka 565-0871, Japan; 9 Department of Pathology, Osaka University Graduate School of Medicine, Osaka 565-0871, Japan; 10 Department of Breast and Endocrine Surgery, Osaka University Graduate School of Medicine, Osaka 565-0871, Japan; 11 Department of Otorhinolaryngology-Head and Neck Surgery, Osaka University Graduate School of Medicine, Osaka 565-0871, Japan; 12 Strategic Global Partnership & X-Innovation Initiative Graduate School of Medicine, Osaka University & Osaka University Hospital, Osaka 565-0871, Japan; 13 Department of Cancer Immunotherapy, Osaka University Graduate School of Medicine, Osaka 565-0871, Japan; 14 Department of Cancer Immunology, Osaka University Graduate School of Medicine, Osaka 565-0871, Japan; 15 Laboratory of Cellular Immunotherapy, World Premier International Immunology Frontier Research Center, Osaka University, Osaka 565-0871, Japan; 16 Department of Cancer Stem Cell biology, Osaka University Graduate School of Medicine, Osaka 565-0871, Japan; 17 Department of Urology, Osaka International Cancer Institute, Osaka 541-8567, Japan; 18 Department of Dermatology, Osaka University Graduate School of Medicine, Osaka 565-0871, Japan; 19 Department of Gynecology, Osaka University Graduate School of Medicine, Osaka 565-0871, Japan; 20 Department of Surgery, Osaka Police Hospital, Osaka 543-0035, Japan; 21 Department of Medical Physics and Engineering, Osaka University Graduate School of Medicine, Osaka 565-0871, Japan; 22 Department of Urology, Osaka University Graduate School of Medicine, Osaka 565-0871, Japan; 23 Department of Hematology and Oncology, Osaka University Graduate School of Medicine, Osaka 565-0871, Japan

**Keywords:** rare cancer, WT1, cancer vaccine

## Abstract

**Simple Summary:**

Therapeutic options for rare cancers are frequently limited and less effective than for common cancers. Therefore, novel therapeutic strategies to treat rare cancers are urgently required. Our clinical study on rare cancers showed that the biweekly WT1 Trio peptide vaccine comprising two WT1-cytotoxic T lymphocyte (CTL)-peptides and one WT1-helper T lymphocyte-peptide induced more robust immune responses targeting WT1 than the weekly WT1-CTL peptide (WT1-235) vaccine. In addition, the safety of WT1 Trio was confirmed without severe treatment-related adverse events, except grade 3 myasthenia gravis-like symptoms observed in a patient with thymic cancer (TC). Fifteen (33.3%) of the 45 patients with recurrent or advanced rare cancers, including malignant glioma, soft-tissue sarcoma, TC, and malignant pleural mesothelioma, achieved stable disease after 3 months of protocol treatment. Therefore, since WT1 is widely overexpressed in rare cancers, WT1-targeted immunotherapy may be a therapeutic strategy for rare cancers.

**Abstract:**

No standard treatment has been established for most rare cancers. Here, we report a clinical trial of a biweekly WT1 tri-peptide-based vaccine for recurrent or advanced rare cancers. Due to the insufficient number of patients available for a traditional clinical trial, the trial was designed for rare cancers expressing shared target molecule WT1. The recruitment criteria included WT1-expressing tumors as well as HLA-A*24:02 or 02:01. The primary endpoints were immunoglobulin G (IgG) antibody (Ab) production against the WT1-235 cytotoxic T lymphocyte (CTL) epitope and delayed-type hypersensitivity (DTH) skin reactions to targeted WT1 CTL epitopes. The secondary endpoints were safety and clinical efficacy. Forty-five patients received WT1 Trio, and 25 (55.6%) completed the 3-month protocol treatment. WT1-235 IgG Ab was positive in 88.0% of patients treated with WT1 Trio at 3 months, significantly higher than 62.5% of the weekly WT1-235 CTL peptide vaccine. The DTH positivity rate in WT1 Trio was 62.9%, which was not significantly different from 60.7% in the WT1-235 CTL peptide vaccine. The WT1 Trio safety was confirmed without severe treatment-related adverse events, except grade 3 myasthenia gravis-like symptoms observed in a patient with thymic cancer. Fifteen (33.3%) patients achieved stable disease after 3 months of treatment. In conclusion, the biweekly WT1 Trio vaccine containing the WT1-332 helper T lymphocyte peptide induced more robust immune responses targeting WT1 than the weekly WT1-235 CTL peptide vaccine. Therefore, WT1-targeted immunotherapy may be a potential therapeutic strategy for rare cancers.

## 1. Introduction

Rare cancers comprise a diverse collection of less common cancers. According to the National Cancer Institute definition [1], all but 11 cancer types, including breast, lung, prostate, colon, and rectal cancers, are classified as rare in American adults [2]. Although the number of patients with cancer is small, 15–25% are diagnosed with rare cancers [3,4]. Compared with the more common malignancies, the 5-year survival rate is worse in patients with rare cancers. Several essential factors, including limited well-established therapies and delayed diagnosis, contribute to poor survival. Therefore, since treatment options for rare cancers are frequently more limited and less effective than those for more common cancers, novel therapeutic strategies to treat rare cancers are urgently needed. 

The *WT1* gene was initially isolated as a tumor suppressor gene responsible for Wilms’ tumor, which is a pediatric renal neoplasm [5]. However, WT1 has oncogenic functions [6,7,8,9,10] and is overexpressed in leukemia [11] and various types of solid tumors, including lung [12], colorectal [13], pancreatic [14], malignant gliomas [15], and bone and soft-tissue sarcomas [16]. WT1-targeted immunotherapy has been considered a promising therapeutic strategy for various cancers owing to its tumor-specific expression and high immunogenicity [17,18,19]. Consequently, we and others have demonstrated the clinical utility of WT1-targeted immunotherapies in multiple forms, including a WT1 peptide cancer vaccine [20,21,22,23], WT1 peptide-pulsed dendritic cell vaccine [24], or WT1 mRNA-electroporated dendritic cell vaccine [25], as well as WT1-specific T cell receptor-transduced T-cell therapy [26,27].

We conducted multiple clinical trials using the HLA-A*24:02 binding cytotoxic T lymphocyte (CTL) peptide WT1-235, which showed an association of favorable prognosis with a positive delayed-type hypersensitivity (DTH) skin test for the WT1-235 peptide [28,29,30] and production of WT1-235 peptide immunoglobulin G (IgG) antibody (Ab) [29,30]. As the class switch from IgM to IgG requires the help of helper T cells, the association between WT1 peptide IgG Ab production and favorable prognosis suggests the importance of cluster of differentiation 4 (CD4^+^) T cells in WT1 peptide vaccines. We identified WT1-332 as an HLA class II-binding helper T lymphocyte (HTL) helper peptide and demonstrated that it enhanced WT1 CTL responses in vitro [31]. Furthermore, we recently demonstrated that a combination of helper peptides enhances the intratumoral infiltration of lymphocytes in a tumor-bearing mouse model [32]. Therefore, based on these preclinical data, we conducted a clinical trial of the cocktail vaccine for recurrent glioblastoma multiforme, a weekly combinatorial vaccination of CTL peptide, and an HTL peptide vaccine at 2-week intervals. This clinical trial showed the clinical safety of a combination of WT1 CTL and HTL peptides [33]. The cocktail vaccine induced Th1-type, WT1-235-specific CD8^+^ CTLs, more efficiently than the WT1-235-CTL peptide vaccine at 4–7 weeks after the start of vaccination. In patients receiving the cocktail vaccine, the frequency of WT1 CD8^+^ CTLs correlated with that of WT1-332-specific CD4^+^ T cells [34]. These findings show that the HLA class II-binding HTL helper peptide WT1-332 can enhance the anti-tumor effects of the WT1-235 CTL vaccine in clinical settings.

Recent advances in understanding oncogenic molecular abnormalities and the ability to target them have provided a therapeutic spectrum across tumor types, including molecular targets shared by various tumor tissues [35]. Since all WT1-expressing tumors are targets of WT1-specific CTLs, WT1 may be a common target antigen for rare cancers, and WT1-targeting immunotherapy could be applied to rare cancers expressing WT1. Here, we report a clinical trial of a WT1 Trio peptide vaccine, which comprises two WT1 CTL peptides, WT1-126 and WT1-235, and an HTL peptide, WT1-332. In this clinical trial, we evaluated whether the WT1 Trio peptide vaccine enhanced the DTH response and IgG Ab production in patients with recurrent or advanced rare cancers where no standard treatment has been established as the primary endpoints. Additionally, we evaluated safety, clinical efficacy, and other immune responses as the secondary endpoints.

## 2. Materials and Methods

### 2.1. Peptides

The Good Manufacturing Practice grade WT1-235 (CYTWNQMNL) [20,21,22,23], WT1-126 (RMFPNAPYL) [17], and WT1-332 (KRYFKLSHLQMHSRKH) [33] for HLA-A*24:02, HLA-A*02:01, and HLA class II, respectively, were synthesized at the Peptide Institute (Ibaraki, Osaka, Japan). The biophysical properties of the peptides are presented in Table S1.

### 2.2. Treatment 

**Figure 1:**
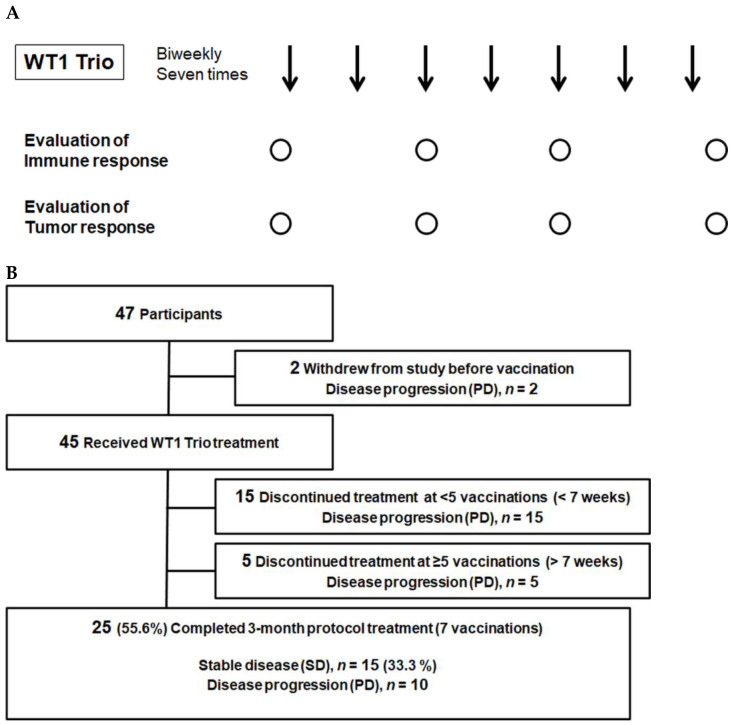
Clinical study design and patient flow chart. (**A**) Clinical trial schema. Arrows, administration of WT1 Trio; circles, Collection of blood samples and image testing. (**B**) Flowchart of the enrolled patients. PD, progressive disease; SD, stable disease.

Patients were recruited between May 2017 and December 2018. The eligibility criteria included the following: histologically confirmed rare cancers unamenable to potentially curative therapies. In addition, WT1 protein expression in tumor cells, HLA-A*24:02 or 02:01 positivity, the age range of 16–85 years, and good organ function were also assessed. The cancers included malignant glioma (grade III anaplastic astrocytoma and grade IV glioblastoma multiforme), soft tissue sarcoma, malignant pleural mesothelioma (MPM), thymic cancer (TC), thymoma, head and neck mucosal malignant melanoma, ophthalmic malignant melanoma, head and neck mucoepidermoid carcinoma, olfactory neuroblastoma, cancer of the external auditory canal, cancer of the small intestine, salivary gland cancer, testicular cancer, penile cancer, adrenal cancer, Merkel cell carcinoma, and cutaneous lymphoma. As previously described, WT1 protein expression was immunohistochemically assessed [8]. The WT1 Trio peptide vaccine comprised two WT1 CTL peptides, WT1-126 and WT1-235, and an HTL peptide, WT1-332, emulsified with Montanide ISA51 adjuvant (Sepic, Paris, France). We administered 2 mg of each of the three peptides emulsified with Montanide ISA51 adjuvant (Sepic, Paris, France) seven times at 2-week intervals (Figure 1A). The trials were registered as UMIN000023579 (registered on 10 September 2016) in the UMIN Clinical Trials Registry. The ethical review board of the Faculty of Medicine, Osaka University, approved this study (ID 15605, date of approval: 7 September 2016). Furthermore, written informed consent was obtained from all participants at Osaka University Hospital. These clinical trials were one-armed; therefore, they did not adhere to the CONSORT statement. We conducted our study following the Japanese Ethical Guidelines for Medical and Biological Research Involving Human Subjects. The procedure is audited externally.

### 2.3. Evaluation of the Safety and Clinical Efficacy 

Anti-tumor effects were assessed by determining the response of target lesions on computed tomography scans according to the Response Evaluation Criteria in Solid Tumors v1.1 [36]. Safety was assessed by monitoring and recording adverse events (AEs), vital signs, clinical chemistry, and hematology. AEs were graded according to the Common Terminology Criteria for Adverse Events v4.0 [37].

### 2.4. Collection of Samples for Immune Monitoring

A written informed consent for immune monitoring was obtained from the participants at Osaka University Hospital. The ethical review board of the Faculty of Medicine, Osaka University, approved this study (ID:13110 and 11293, date of approval: 17 October 2013 and 15 June 2012). Sera were obtained from patients with written informed consent at Osaka University Hospital at the indicated time points (Figure 1A). Serum samples were stored at −20 °C until use. Furthermore, blood samples were obtained from the patients to collect peripheral blood mononuclear cells (PBMC). PBMCs were isolated from heparinized whole blood using the standard Ficoll-Paque separation method and cryopreserved in liquid nitrogen until further use. 

### 2.5. DTH Skin Test

WT1 DTH skin tests were performed monthly during the protocol treatment. Briefly, 10 μg of each WT1 peptide, WT1-126, WT1-235, and WT1-332, separately dissolved in saline, and saline alone (control) was intradermally injected into the forearm of the skin. DTH-positivity was defined as erythema ≥ 2 mm in diameter measured 48 h after injection.

### 2.6. Enzyme-Linked Immunosorbent Assay (ELISA) 

WT1 epitope-specific IgG Abs in the serum were measured using ELISA, as described previously [29,38]. WT1 peptides or citrate (negative control) were immobilized on the bottom surface of each well using the MK100 Peptide Coating Kit (Takara, Shiga, Japan) according to the manufacturer’s instructions. After blocking, patient sera diluted 1:100 were added to each well and incubated at 4 °C overnight. All serum samples were measured in duplicates. After washing, horseradish peroxidase (HRP)-conjugated rabbit anti-human IgG Ab (Santa Cruz Biotechnology, Santa Cruz, TX, USA) diluted 1:2000 in TBST was added to each well and incubated. After washing, HRP-conjugated goat anti-rabbit IgG, diluted 1:5000 in TBST, was added and incubated. Furthermore, bound WT1 epitope-specific IgG Abs were colorimetrically detected using TMB substrate (KPL, Baltimore, MD, USA). Absorbance was measured at 450 nm using a microplate reader (MULTISKAN FC, Thermo Fisher Scientific, Waltham, MA, USA). The Ab titer for each serum sample was determined as the average absorbance value of duplicate wells after subtracting the absorbance value of the negative control well. The cutoff levels for the positivity of WT1-235 IgG, WT1-126, and WT1-332 IgG were set at 0.15 based on the absorbance values of the mean + 3SD from five independent assays in negative control serum samples.

### 2.7. Enzyme-Linked Immunospot (ELISPOT) Assay

As reported previously, the ELISPOT assay was performed [38,39]. Briefly, after hydrophilization, the bottom membrane of each well in a 96-well filtration plate (Merck) was incubated with capture Ab, anti-human interferon-gamma (IFN-γ) monoclonal Ab (anti-human IFN-γ mAb 1-D1K; MABTECH, Cincinnati, OH, USA, #3420-3-250, final concentration: 15 μg/mL in phosphate-buffered saline [PBS]), anti-human tumor necrosis factor-alpha (TNF-α) monoclonal Ab (anti-human TNF-α mAb TNF3/4; MABTECH, #3510-3-250, final concentration: 7.5 μg/mL of PBS), or anti-human interleukin 10 (IL-10) monoclonal Ab (anti-human IL-10 mAb; final concentration, 15 µg/mL in PBS; cat. no. 3430-3-250; Mabtech AB), at 4 °C overnight. After blocking the membranes, thawed PBMCs suspended in fetal bovine serum (FBS)-free RPMI1640 medium (5 × 10^4^ cells per 100 μL) were seeded in each well in triplicate, stimulated with the antigen peptide at a final concentration of 10 μg/mL, and incubated at 37 °C for 48 h. After removing the cell suspension, each membrane was incubated with the corresponding detection Abs in PBS containing 1% bovine serum albumin and 0.05% tween-20, and a biotinylated anti-human IFN-γ monoclonal Ab (MABTECH, #3420-6-250, final concentration 1.3 μg/mL), a biotinylated-anti-human TNF-α monoclonal Ab (TNF5, MABTECH, #3510-6-250, final concentration of 1.5 μg/mL), or a biotinylated anti-human IL-10 monoclonal Ab (final concentration, 1.3 µg/mL; cat. no. 3430-6-250; Mabtech AB) was subsequently incubated with the sample at 4 °C overnight. After washing with PBS, each membrane was incubated with alkaline phosphatase-conjugated streptavidin (MABTECH, #3310-8, diluted 1:500 with 0.05% PBST) at room temperature for 1 h. After washing both sides of the membranes, the spots were colored using a BCIP/NBT solution (Nacalai Tesque, Kyoto, Japan), followed by washing with deionized water. Furthermore, after drying at 4 °C overnight, the membranes were punched out using an acrylic device, “ELI 8” (Create Ltd., Osaka, Japan). The membranes were scanned at a resolution of 1200 dpi. The generated digital images were analyzed for spot counting with the assistance of particle analysis using the NIH ImageJ software (version 1.50i; National Institutes of Health, Bethesda, MD, USA). WT1 antigen-specific IFN-γ production/secretion by PBMCs was described as the antigen-specific IR index as follows: (number of spot-forming cells in antigen-stimulated test conditions)/(number of spot-forming cells in antigen-free control conditions). The cutoff level for positive detection of antigen-specific cytokine production/secretion was 1.0 in the immune response index (IR index).

### 2.8. Statistical Analysis

Differences in WT1-235 IgG, WT1-126 IgG, and WT1-332 IgG Ab titers were analyzed using Welch’s *t*-test. Differences in the positivity rates of WT1-235 IgG, WT1-126 IgG, and WT1-332 IgG Abs and DTH skin reaction to WT1-126 or WT1-235 CTL epitopes between the WT1-235 CTL peptide vaccine and WT1 Trio at the end of the treatment protocol were analyzed using Fisher’s exact probability test. In addition, the association between immune factors before vaccination with the WT1 Trio and clinical outcomes in patients with malignant glioma was evaluated using Fisher’s exact probability test. The significant difference between Welch’s *t*-test and Fisher’s exact probability test was set at *p* < 0.05. The statistical analysis was performed using the Excel Statcel4 add-in software (OMS Publishing Ltd., Saitama, Japan).

## 3. Results

### 3.1. Patient Characteristics

This clinical trial included 80 participants. WT1 expression in tumor cells and HLA-A was examined after obtaining consent for testing. WT1 expression was confirmed by immunostaining in 73 (91.3%) of the 80 patients analyzed. HLA-A was adapted for 55 (68.8%) of the 80 participants. The enrolled patients included 30 males and 17 females, aged 18–81 (median 55 years). There were 23 malignant gliomas, nine soft tissue sarcomas, five TCs, six MPMs, one bronchial mucoepidermoid carcinoma, one salivary gland cancer, one thymoma, and one olfactory neuroblastoma (Table 1). Soft tissue sarcomas included two leiomyosarcomas, two rhabdomyosarcomas, two synovial sarcomas, one liposarcoma, one myxofibrosarcoma, and one solitary fibrous tumor. 

Number of patients who regularly received steroids and NSAIDs at the time of vaccination initiation is indicated.

The WT1 Trio vaccine was administered following a treatment-free period of at least 4 weeks. We administered one or more WT1 tripeptide vaccines to 45 out of 47 patients. However, 20 patients dropped out due to tumor progression; a total of 25 patients completed the 3-month protocol treatment. Of the 20 dropouts, 15 and 5 dropped out in the first (i.e., before the 7th week) and second (i.e., after the 7th week) half of the protocol treatment period, respectively (Figure 1B).

### 3.2. Immunological Responses

Here, the primary endpoints were the production of the WT1-235 IgG Ab and the DTH reaction to the WT1-235 peptide. This is because both endpoints have been shown to correlate with prognosis in previous studies [28,29,30].

**Figure 2:**
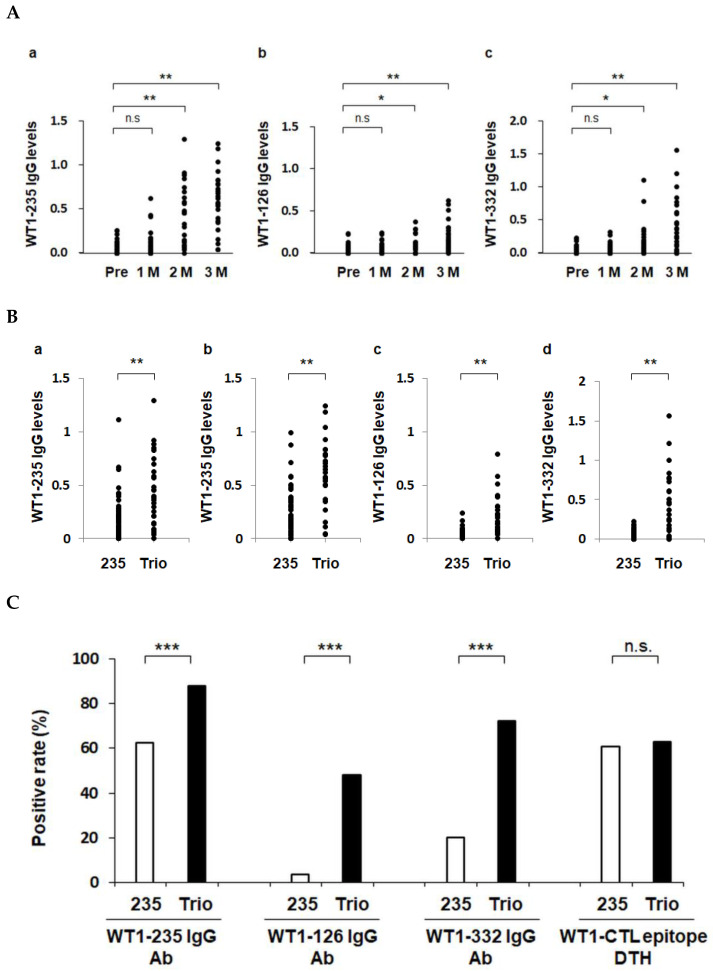
Enhanced induction of WT1-specific immune responses by WT1 Trio. Serum IgG antibody levels against WT1-126, WT1-235, and WT1-332 epitopes were measured using ELISA. *, *p* < 0.05; **, *p* < 0.01, and ***, *p* < 0.001. n.s., not significant. (**A**) Induction of IgG responses during treatment with WT1 Trio. (**B**) Serum IgG levels in the WT1-235 CTL peptide vaccine (235) and the WT1 Trio peptide vaccine (Trio) were compared. Serum IgG levels at 2 months (**a**) and 3 months (**b**–**d**). (**C**) Positive rates of the primary endpoints: serum IgG antibody against the three WT1 epitopes and DTH against WT1-CTL epitopes (WT1-235 and WT1-126).

The serum levels of IgG Abs against the three antigenic epitopes WT1-235, WT1-126, and WT1-332 were measured using ELISA before and at 1, 2, and 3 months (1 M, 2 M, and 3 M) after the start of vaccination. All three serum Ab levels were significantly higher at 2 M and 3 M than those before vaccination, confirming the induction of antigen epitope-specific IR (Figure 2A). 

Next, we compared the biweekly WT1 Trio and weekly WT1-235 CTL peptide vaccines (Figure S1) regarding their induction of IgG Ab production against the target epitope WT1-235. Patients treated with the WT1-235 CTL peptide vaccine included 43 and 13 with malignant glioma [29] and non-small-cell lung cancer, respectively. Regarding Ab levels and positivity rates, WT1 Trio induced the production of WT1-235 IgG Abs more robustly than the WT1-235 peptide vaccine. We further compared the WT1 Trio and WT1-235 CTL peptide vaccines in terms of their induction of IgG Ab production against the WT1-126 and WT1-332 epitopes. Serum levels of WT1-126 and WT1-332 IgG Abs in patients treated with WT1 Trio were significantly higher than those in the WT1-235 CTL peptide vaccine at 3 M of vaccination. These results show that the WT1 Trio has more potential to induce the production of IgG Abs than the WT1-235 CTL peptide vaccine (Figure 2B,C).

We evaluated CTL epitope-specific DTH responses in 27 patients treated with WT1 Trio, for which data were available both before vaccination and 1 M after the vaccination began. In patients treated with WT1 Trio, 17 of the 27 (62.9%) were positive for the DTH skin test for HLA-matched CTL peptide. In patients treated with the WT1-235 vaccine in the control group, 34 of 56 (60.7%) patients were positive for the DTH skin test. The positivity rates were not significantly different between the WT1 Trio and WT1-235 CTL peptide vaccines (Figure 2C).

**Figure 3:**
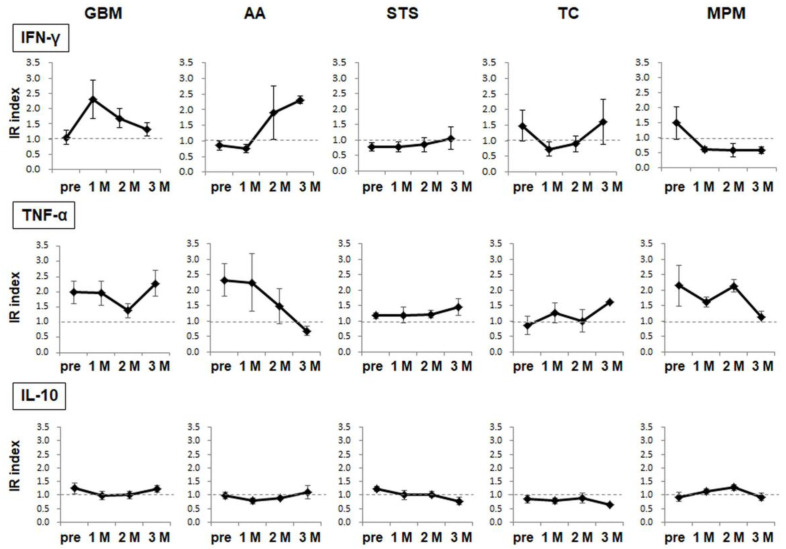
WT1-specific cellular immune responses. The target epitope WT1-235-specific cytokine production/release from PBMCs was analyzed using the ELISPOT assay. The results are indicated in the IR index for each cytokine. The circles and error bars indicate the average and standard error, respectively.

We further analyzed the cellular immune responses to the target antigen WT1-235 using the ELISPOT assay. The production and secretion of three cytokines from PBMCs collected before vaccination and 1 M, 2 M, and 3 M after the start of vaccination. WT1-235-specific cellular immune responses were induced in different patterns, depending on the disease group. Regarding WT1-235-specific IFN-γ production, the IR index increased in patients with GBM and AA after the initiation of the administration, although the timing after the start of vaccination differed. In patients with soft-tissue sarcoma (STS), the IR index increased slowly and became weakly positive at 3 months. In contrast, the IR index, which was positive before vaccination, decreased after the start of vaccination in patients with TC and MPM. For WT1-235-specific TNF-α production, the IR index was positive in patients with GBM, AA, or MPM before vaccination. However, the IR index declined after the start of administration in all three diseases and recovered at 3 M in GBM. Conversely, it continued to decline during the 3 months of protocol treatment in AA and MPM. The IR index of IL-10, which was a type 2 T helper cell (Th2)-type cytokine, was constant at approximately 1.0 in all diseases, and the antigen-specific Th2-type cellular immune responses were weak during the 3-month treatment period. In conclusion, we found that cellular immune responses changed dynamically after the start of the WT1 Trio vaccination, and that their mode differed depending on the specific disease (Figure 3).

### 3.3. Safety

Safety was analyzed in all 45 patients who received one or more doses of WT1 Trio. The AEs observed during the WT1 Trio treatment period are presented in Table 2. Skin reactions at the vaccination site (grade 1) were observed in 30 (66.7%) of the 45 patients. In addition, it was found that interstitial pneumonia (grade 1) was probably related to the WT1 Trio in a patient with MPM (Pt no. 17) and that myasthenia gravis-like symptoms (grade 3) were possibly associated with the WT1 Trio in a patient with TC (Pt no. 28). Two deaths were recorded in this study. One patient with synovial sarcoma died during the protocol treatment (Pt no. 13), and another with TC died within 30 days of the last vaccination with the WT1 Trio (Pt no. 45). Both deaths were attributed to disease progression (PD). Notably, no grade 4 or 5 AEs related to the WT1 Trio were observed (Table 2).

### 3.4. Clinical efficacy

As outlined above, the WT1 Trio vaccine was administered following a treatment-free period of at least 4 weeks. The previous treatment regimens of the patients who were administered WT1 Trio are summarized in Table 3.

#### 3.4.1. Malignant Glioma

Here, 15 and 8 patients with glioblastoma multiforme and anaplastic astrocytoma, respectively, were enrolled. Before vaccination, one patient with glioblastoma multiforme dropped out because of PD. Seven (50.0%) of the 14 patients with glioblastoma multiforme completed the 3-month protocol treatment. Four of the seven patients who dropped out stopped the WT1 Trio until 7 weeks due to PD. Four patients achieved stable disease after 3 months of vaccination. Two (25.0%) of the eight patients with anaplastic astrocytoma completed the 3-month protocol treatment. Both patients achieved stable disease within 3 months of vaccination. Five of the six patients who dropped out stopped the WT1 Trio until 7 weeks due to PD.

#### 3.4.2. STS

Before vaccination, one patient with STS dropped out because of PD. Five (62.5%) of the eight patients completed the 3-month protocol treatment. All three patients who dropped out stopped the WT1 Trio vaccine until 7 weeks due to PD. Two (25.0%) patients achieved stable disease after 3 months of vaccination.

#### 3.4.3. MPM

Here, six patients were enrolled. Four (66.7%) of the six patients completed the 3-month protocol treatment. Two patients who dropped out stopped the WT1 Trio until 7 weeks due to PD. Three (50.0%) patients achieved stable disease after 3 months of vaccination.

#### 3.4.4. TC

Here, five patients were enrolled. Three (60.0%) of the five patients completed the 3-month protocol treatment. Unfortunately, two patients dropped out due to PD during the protocol treatment period. One (20.0%) patient achieved stable disease after 3 months of vaccination.

#### 3.4.5. Others

Here, four patients with four different tumors (olfactory neuroblastoma, broncho-mucoepidermal carcinoma, salivary adenocarcinoma, and thymoma) were enrolled. All patients completed the 3-month protocol treatment. Patients with olfactory neuroblastoma, broncho-mucoepidermal carcinoma, and salivary adenocarcinoma achieved SD. The tumor response in a patient with thymoma was PD.

### 3.5. Association between Immune-Related Factors before the Vaccination Start and Prognosis

Here, we analyzed the association between immune-related factors before vaccination and clinical outcomes in patients with malignant glioma who received WT1 Trio once or more than once. Immune-related factors included the neutrophil/lymphocyte ratio (N/L ratio), spontaneous IFN-γ, TNF-α, and IL-10 released from PBMCs, as well as WT1-332-specific IFN-γ, TNF-α, and IL-10 released from PBMCs in all patients with malignant glioma and WT1-235-specific IFN-γ and IL-10 released from PBMCs in patients with HLA-A*24:02. Clinical outcomes were assessed by the completion of the 3-month treatment protocol and tumor response during the study. Of these immune-related factors, the group with positive spontaneous IL-10 released from PBMCs had a significantly lower completion rate and a higher rate of tumor progression. In addition, the group with WT1-332-specific IL-10 released from PBMCs tended to have a lower completion rate and a higher rate of tumor progression. When the cutoff level of the WT1-332-specific IL-10 IR index was set as 1.15, the group with a higher WT1-332-specific IL-10 released from PBMCs had a significantly lower completion rate and a higher rate of tumor progression than the group with lower WT1-332-specific IL-10 released from PBMCs (Table 4).

These results may imply that the immunosuppressive environment before vaccination is associated with poor clinical outcomes in patients treated with WT1 Trio.

## 4. Discussion

This clinical study revealed the following findings: (1) WT1, the target antigen of the WT1 Trio cancer vaccine, is highly expressed in the majority of rare cancers. (2) Biweekly administration of the WT1 Trio vaccine led to a more significant enhancement of IgG antibody production against the WT1-235 peptide compared to weekly administration of the WT1-235CTL peptide vaccine. (3) The WT1 Trio vaccine did not enhance CTL epitope-specific DTH, which is regarded as another primary endpoint. (4) The WT1 Trio vaccine is safe for patients without risk of autoimmune predisposition. (5) The WT1-332 HTL epitope-specific, Th2-type cellular immune response experienced prior to vaccination was associated with poor prognosis in WT1 Trio-treated recurrent malignant glioma.

When recruiting patients, we immunohistochemically examined the expression of WT1, the target antigen of the WT1 Trio cancer vaccine. WT1 protein expression in tumor cells was confirmed in most patients recruited for this clinical trial. These include GBM, AA, TC, soft tissue sarcoma, MPM, and olfactory neuroblastoma. Since no standard treatments have been established for most rare cancers, developing novel treatment strategies is urgently required [3,4]. All WT1-expressing tumor cells could be targets of WT1-specific CTLs. The high expression rate of the WT1 protein in tumor cells indicates that cancer immunotherapy targeting WT1 may be a therapeutic strategy for many rare cancers as an immunotherapy against a common target molecule.

We analyzed the production of IgG Abs against the WT1 peptide (the first primary endpoint of this clinical trial) by monitoring the immune responses against WT1, the target antigen of the WT1 Trio cancer vaccine. Abs are produced by activating antigen-specific B cells following cognate interactions with CD4^+^ T cells to help drive B-cell expansion, germinal center formation, and differentiation into Ab-secreting cells [40]. Therefore, the production of IgG Abs serves as an immune monitoring marker that indicates the activation of CD4^+^ T cells and antigen-specific B cell lineages, which play crucial roles in immune responses. Here, we analyzed the ability of WT1 Trio to induce WT1-specific immune responses as a primary endpoint. The enhanced WT1-235 IgG Ab production observed in this study showed that biweekly vaccination with WT1 Trio, which contains two WT1 CTL peptides, WT1-235 and WT1-126, as well as WT1 HTL peptide 332, induced immune responses mediated by helper T cells and WT1-235 epitope-specific B cell lineages more robustly than weekly vaccination with WT1-235 peptide vaccine. 

WT1 Trio cancer vaccine-induced IgG antibody production against WT1 peptides indicates the activation of WT1-specific B-lymphocyte lineage cells. Recent evidence has shown that B cells play a crucial role in tumor cellular immune responses. Several studies have reported that some tumors generate adaptive immune responses in spatially well-organized structures called tertiary lymphoid structures (TLS). TLS has been observed in various types of tumors, including lung [41,42], breast [43,44], and sarcomas [45]. A positive association between the presence of TLS and B lymphocytes and therapeutic responses to immune checkpoint inhibitors has been reported in melanoma [46] and sarcoma [47]. One possible anti-tumor cellular immune mechanism relevant to B cells is antigen presentation. Compared with static B cells expressing scant co-stimulatory molecules, activated B cells have a strong antigen-presenting function that promotes the proliferation and IFN-γ production by CD8^+^ T cells [48]. Furthermore, as a cellular immune mechanism of B cells, it has been reported that B cells can produce and secrete cytokines and may function as helper cells [49]. Interestingly, we found that the WT1 Trio induced the production of IgG Abs against the three administered WT1 epitope peptides. These results indicate the existence of B cells with B cell receptors that recognize the CTL epitopes WT1-235 and WT1-126 and the HTL epitope WT1-332. These WT1-specific B cells are activated by the administered WT1 Trio and may affect clinical outcomes through antigen presentation or immunomodulation of the tumor immune microenvironment via cytokine production and release.

We also analyzed the DTH skin reaction to the WT1 peptide (the other primary endpoint of this clinical trial) by monitoring the immune response against WT1, the target antigen of the WT1 Trio cancer vaccine. We previously reported that DTH against the WT1 peptide correlated with favorable prognosis in WT1 peptide vaccines for patients with pancreatic cancer and glioblastoma multiforme [28,29]. However, the positive rates of DTH in patients receiving WT1 Trio did not differ significantly from those treated with the WT1-235 CTL peptide vaccine. These results show that WT1 Trio enhanced WT1-specific immune responses detected by WT1-235 IgG Ab production, rather than WT1-specific immune responses represented by DTH responses, as compared with the WT1-235 CTL peptide vaccine. Furthermore, these results indicate that the ability of the WT1 Trio to induce aWT1-specific cellular immune response detected by the DTH response is not superior to that of the WT1-235 CTL peptide vaccine. These findings show that the WT1 Trio peptide vaccine could be further improved to induce more robust WT1-specific cellular immune responses for better clinical outcomes.

As a secondary endpoint, we evaluated the safety of biweekly WT1 Trio administration. The WT1 Trio vaccine, which includes a helper peptide (WT1-332), was administered in combination in order to activate WT1-specific CD4^+^ T helper lymphocytes and enhance WT1-specific anti-tumor cellular immune responses. Since CD4^+^ T cells play a crucial role in regulating immune responses [50], their activation induced by the WT1 Trio might enhance immune responses toward the target and other antigens. WT1 protein is expressed in the pleura and renal podocytes in normal adult tissues. However, no treatment-related pleural or renal events were observed in this clinical study. As an AE in WT1-non-expressing tissues, acetylcholine receptor Ab-negative myasthenia gravis-like symptoms (G3) developed in a patient with TC (Pt no. 28) who received four doses of WT1 Trio. The symptoms improved to grade 1 after discontinuation of the WT1 Trio and administration of gamma globulin, implying that an immunological mechanism causes myasthenia gravis-like symptoms. Although myasthenia gravis is a well-known complication of TC as a paraneoplastic syndrome, activation of the general immune system by the WT1 Trio may enhance autoimmune responses in the patient. Therefore, careful considerations should be taken when recruiting patients at risk of autoimmune predisposition.

The observation period of this clinical study was 3 months. In a subsequent clinical trial, we will continue administering the WT1 Trio peptide vaccine to patients in whom the WT1 Trio is considered to slow tumor growth. In addition, future studies are required to examine the long-term induction and maintenance of WT1-specific immune responses, safety, and clinical efficacy in patients treated with WT1 Trio.

In addition to the primary endpoints investigated through immune monitoring, we examined the changes in WT1-specific cellular immune responses using an ELISPOT assay. ELISPOT assays revealed that WT1-specific cellular immune responses differed significantly in patients treated with WT1 Trio regarding robustness before vaccination, mode of immune response induction after vaccination, and maintenance of the cellular immune responses after their induction. These results indicate that each patient’s optimal dose and vaccination schedule may vary according to their immunological status. Therefore, individualized vaccination may be required based on biomarkers that can predict clinical efficacy before vaccination and monitor anti-tumor immune responses after the start of vaccination. In addition, the ELISPOT assay showed a decline in WT1-235 epitope-specific IFN-γ production from PBMCs one month after WT1 Trio initiation; this decline persisted throughout the 3-month treatment protocol in MPM cases. However, WT1-235Ab production increased in all four MPM cases that completed the treatment protocol. The DTH skin test demonstrated positive results in two of the four cases examined. Thus, it is evident that WT1 Trio induced WT1-specific immune responses in these cases. This discrepancy could be explained by the fact that most of the enrolled cases with MPM had large tumor burdens. This raises the possibility of migration of many activated WT1-235-specific immune cells from peripheral blood to the tumor site, which ultimately could have led to a decrease in WT1-235-specific cellular immune responses in PBMCs. On the other hand, WT1 Abs reflect the systemic immune responses of the B lymphocyte lineage, and the WT1 DTH skin test reflects the immune responses of the immune cells that infiltrated the skin tissue from peripheral blood. These differences in monitoring targets may have led to a discrepancy in the assessment of immune induction. Since it is impossible to monitor local immune dynamics in the tumor in real time, it is necessary to comprehensively evaluate the immune responses based on the characteristics of immune monitoring markers.

Predicting treatment response is essential for patient selection. In this study, we analyzed the association between WT1-specific cellular immune responses before vaccination and prognosis. This study showed that spontaneous IL-10 secretion and HTL epitope WT1-332-specific IL-10 secretion from PBMCs before vaccination were associated with a poor clinical prognosis in patients with recurrent malignant glioma treated with the WT1 Trio cancer vaccine. The inhibitory cytokine IL-10 acts on immune cells, including T cells and macrophages, directly suppressing cell activation and attenuating the antigen-presenting ability of macrophages, thereby lowering immune responses [51]. Therefore, it is reasonable to conclude that immunosuppressive immune status before vaccination is associated with a poor clinical prognosis. Another important point regarding epitope-specific IL-10 production is that HTL epitope-specific IL-10 production is significantly associated with a poor clinical prognosis. The WT1 Trio is a therapeutic vaccine that induces WT1-specific CTLs by combining the HTL epitope WT1-332 peptide with the CTL epitopes WT1-126 and WT235. However, if the WT1-332 peptide strongly induces immunosuppressive IL-10 production/secretion by HTLs, it may have a negative impact on the anti-tumor immune responses. Therefore, the two immune markers identified in this study may be helpful for prognostic prediction before the start of vaccination. However, further studies are required to examine whether they can be used as markers for patient selection.

## 5. Conclusions

In this study, we conducted a clinical trial evaluating a biweekly WT1 tri-peptide-based vaccine for recurrent or advanced rare cancers. Immunological analysis showed that the biweekly WT1 Trio vaccine containing the WT1-332 helper T lymphocyte peptide induced more robust immune responses targeting WT1 as compared with the weekly WT1-235 CTL peptide vaccine. The safety of the WT1 Trio vaccine was confirmed due to a lack of severe treatment-related adverse events, with the exception of grade 3 myasthenia gravis-like symptoms observed in a patient with thymic cancer. Fifteen (33.3%) patients achieved stable disease after 3 months of treatment. Therefore, we conclude that WT1-targeted immunotherapy may be a potential therapeutic strategy for rare cancers.

## Figures and Tables

**Table 1:** Patient characteristics.

Disease	Pt no.	Age (Median)	Sex		HLA-A			Steroid	NSAID
			M	F	24:02	02:01	Both		
Glioblastoma multiforme (GBM)	15	26–75 (45)	8	7	9	4	2	5	1
Anaplastic astrocytoma (AA)	8	28–72 (57)	7	1	7	0	1	3	0
Soft-tissue (STS)	9	18–81 (61)	4	5	5	4	0	0	3
Thymic cancer (TC)	5	43–78 (58)	3	2	3	2	0	0	3
Malignant pleural mesothelioma (MPM)	6	53–76 (72)	5	1	3	1	2	0	4
Others									
Total	47	18–81 (55)	30	17	28	14	5	8	13

Pt no.: number of patients treated with WT1 Trio; M, Male; F, Female; HLA-A, number of enrolled patients with HLA-A:24:02 or 02:01; Both, patients with both HLA-A*24:02 and 02:01; Steroid and NSAID, number of patients who received steroid and non-steroid anti-inflammatory drug (NSAID) at the time of vaccine initiation.

**Table 2:** Adverse events.

	All AE		TRAE	
	Any Grade	Grade ≥ 3	Any Grade	Grade ≥ 3
Death within 30 days from vaccination	2	2	0	0
Myasthenia gravislike symptoms	1	1	1	1
Vaccination site skin reaction	30	0	30	0
Pneumonitis	1	0	1	0
Anemia	28	0	0	0
Increased hemoglobin	4	0	0	0
Thrombocytopenia	4	0	0	0
Leukopenia	6	0	0	0
Lymphocytopenia	36	9	0	0
Increased ALT	13	0	0	0
Increased ALP	15	0	0	0
Increased bilirubin	3	0	0	0
Increased creatinine	6	0	0	0
Hyponatremia	14	1	0	0
Hypernatremia	2	0	0	0
Hypokalemia	5	0	0	0
Hyperkalemia	7	0	0	0
Hypoalbuminemia	18	0	0	0
Dehydration	1	0	0	0
Infectious mononucleosis	1	0	0	0
Dorsal cellulitis	1	0	0	0
Bacterial pneumonia	1	1	0	0

AE, adverse events; TRAE, treatment-associated adverse events.

**Table 3:** Previous treatment regimens.

	Pt no.	RO	Rad-TMZ	Carm. Wafer	Bev	ICI	Chemo One	Other
GBM	14	14	14	6	6	2	1	1
AA	8	7	7	1	5	0	1	2
	**Pt no.**	**RO**	**Rad**	**Chemo One**	**Chemo ≥ 2**	**ICI**	**Other**	
STS	8	7	5	2	4	0	1	
TC	5	2	3	1	4	0	0	
MPM	6	0	0	0	6	1	1	
Others	4	3	3	2	1	1	1	

Pt no.: number of patients treated with WT1 Trio; RO, surgical resection; Rad-TMZ, radiation and temozolomide combined therapy; Carm. Wafer, carmustine wafer; Bev, bevacizumab; ICI, immune checkpoint inhibitor; Chemo one, chemotherapy with one regimen; Chemo ≥ 2, chemotherapy with two or more regimens.

**Table 4:** Associations between immune factors before vaccination and clinical outcomes in patients with malignant glioma.

Immune Factors before Vaccination	Group	Pt no.	Protocol Treatment			Tumor Response		
			COMPL	DROP	*p* Value	SD	PD	*p* Value
N/L ratio	high (≥3)	15	7	8	0.867	4	11	0.448
	low	7	3	4		3	4	
spon. IFN-γ	high (≥400 spots)	7	2	5	0.217	2	5	0.525
	low	14	8	6		6	8	
spon. TNF-α	high (≥140 spots)	15	5	10	0.038 *	5	10	0.477
	low	6	5	1		3	3	
spon. IL-10	high (≥200 spots)	7	1	6	0.031 *	1	6	0.112
	low	14	9	5		7	7	
WT1-332 IFN-γ	pos (IR index > 1.0)	8	3	5	0.466	2	6	0.332
	neg	13	7	6		6	7	
WT1-332 TNF-α	pos (IR index > 1.0)	8	4	4	0.864	4	4	0.378
	neg	13	6	7		4	9	
WT1-332 IL-10	pos (IR index > 1.0)	12	4	8	0.130	3	9	0.154
	neg	9	6	3		5	4	
WT1-332 IL-10	high (IR index > 1.15)	7	0	7	0.002 **	0	7	0.011 *
	low	14	10	4		8	6	
WT1-235 IFN-γ	pos (IR index > 1.0)	7	2	6	0.627	2	5	0.682
	neg	10	4	5		2	8	
WT1-235 TNF-α	pos (IR index > 1.0)	15	5	10	n.d.	4	11	n.d.
	neg	2	1	1		0	2	
WT1-235 IL-10	pos (IR index > 1.0)	10	4	6	0.627	2	8	0.682
	neg	7	2	5		2	5	

Pt no.: number of patients treated with WT1 Trio; COMPL, completion; DROP, Dropout; Spontaneous and WT1 epitope-specific cytokine release from PBMCs were using an ELISPOT assay. Spon., spontaneous; Pos, positive; neg, negative. SD, stable disease; PD, progressive disease. *, *p* < 0.05; **, *p* < 0.01. n.d., not done.

## Data Availability

The de-identified data supporting this manuscript are available upon reasonable request to the corresponding author.

## Supplementary Materials

The following supporting information can be downloaded at: https://www.mdpi.com/article/10.3390/cancers15020393/s1, Table S1: Biophysical properties of WT1 peptides; Figure S1: WT1 peptide data.
