# Supplementary material for: WT1 Trio Peptide-Based Cancer Vaccine for Rare Cancers Expressing Shared Target WT1
Source: Cancers (Basel). 2023 Jan 6;15(2):393. doi: 10.3390/cancers15020393 (PMC9857088; doi:10.3390/cancers15020393)
Supplement: Supplementary file 1 [file cancers-15-00393-s001.zip › cancers-2100591-supplementary.pdf]

cancers

MDPI

Supplementary Materials

WT1 Trio Peptide-Based Cancer Vaccine for Rare Cancers Expressing Shared Target WT1

Yusuke Oji, Naoki Kagawa, Hideyuki Arita, Norifumi Naka, Ken-ichiro Hamada, Hidetatsu Outani, Yasushi Shintani, Yoshito Takeda, Eiichi Morii, Kenzo Shimazu, Motoyuki Suzuki, Sumiyuki Nishida, Jun Nakata, Akihiro Tsuboi, Miki Iwai, Sae Hayashi, Rin Imanishi, Sayaka Ikejima, Mizuki Kanegae, Masahiro Iwamoto, Mayu Ikeda, Kento Yagi, Haruka Shimokado, Hiroko Nakajima, Kana Hasegawa, Soyoko Morimoto, Fumihiro Fujiki, Akira Nagahara, Atsushi Tanemura, Yutaka Ueda, Tsunekazu Mizushima, Masato Ohmi, Takayuki Ishida, Manabu Fujimoto, Norio Nonomura, Tadashi Kimura, Hidenori Inohara, Seiji Okada, Haruhiko Kishima, Naoki Hosen, Atsushi Kumanogoh, Yoshihiro Oka and Haruo Sugiyama

Table S1. Biophysical properties of WT1 peptides.

**Biophysical property**	**WT1-126**	**WT1-235**	**WT1-332**
Sequence	RMFPNAPYL	CYTWNQMNL	KRYF-KLSHLQMHSRKH
Number of residues	9	9	16
Modification	None	None	None
Iso-electric point	pH 9.55	pH 2.91	pH 11.52
Hydrophobicity (%)	44	44	25
Net charge at pH 7	1	−0.1	5.3
Mass spectrometry (m/z)	1108.4	1172.4	2095.2

WT1-126

MS results

株式会社ペプチド研究所
彩都研究所
〒567-0085 大阪府茨木市彩都あさぎ7丁目2番9号
Phone:(072)643-4411 Fax:(072)643-4422
PEPTIDE INSTITUTE, INC.
SAITO RESEARCH CENTER
7-2-9 SAITO-ASAGI, IBARAKI-SHI, OSAKA 567-0085, JAPAN
Phone:81-72-643-4411 Fax:81-72-643-4422

PEPTIDE

品質管理責任者

試験検査報告書

２０１１年４月１９日

品名	WT1-126 ペプチド
構造	Arg-Met-Phe-Pro-Asn-Ala-Pro-Tyr-Leu
ロット番号	610328
製造日	2011 年 3 月 31 日
容量・数量	1 g × 10
検査年月日	2011 年 4 月 4 日～2011 年 4 月 11 日

検査項目	検査結果
1. 性状	白色粉末
2. 純度試験*1	99% （規格:95 %以上）
3. 構成アミノ酸*2	構成アミノ酸に由来する 8 種のアミノ酸ピークを認めた.<br>Leu を 1 としたときのモル比率<br>Asx(1) 1.009, Ala(1) 1.002, Met(1) 0.996, Leu(1) 1.000, Tyr(1) 0.999, Phe(1) 1.000, Arg(1) 1.009, Pro(2) 2.088<br>Leu の回収率 82.64%
4. 酢酸*3	11.66%
5. 水分*4	2.74%
6. 元素分析	実測値 C, 52.55; H, 7.24; N, 14.03 %<br>$C_{52}H_{77}N_{13}O_{12}S_1 \cdot 2CH_3COOH \cdot 3H_2O$ からの計算値 C, 52.45; H, 7.15; N, 14.20 %
→ 7. 質量分析 (ESI-MS)	実測値: *m/z* 1108.4 （計算値: $[M+H]^+$exact =1108.554）<br>results calculated value
8. 残留溶媒*3	ジイソプロピルエーテル:50ppm 以下(実施した分析条件で検出限界以下)<br>アセトニトリル :40ppm 以下(実施した分析条件で検出限界以下)
9. エンドトキシン試験*5	0.02EU/mg 以下
10. 微生物試験	好気性細菌数: 10cfu/0.1g 以下<br>真菌数: 10cfu/0.1g 以下

備考
HPLC チャート 2 枚添付
*1 日局 15 一般試験法, 液体クロマトグラフィーに準じて行う.
カラム:YMC Pack ODS-A, 4.6 mm I.D. × 150 mm
溶離液:0.1 % TFA
グラディエント CH_3CN 10 % → 60 %(25 min)
流 速:1.0 mL/min, カラム温度:25℃, 検 出:220 nm
*2 加水分解条件: 6 mol/L 塩酸(2%フェノール添加), 110℃, 22 時間
*3 日局 15 一般試験法, ガスクロマトグラフィーに準じて行う.
*4 日局 15 一般試験法, 水分測定法(電量滴定法)に準じて行う.
*5 日局 15 一般試験法, エンドトキシン試験法(光学的測定法 (1)比濁法)に準じて行う.

WT1-126

HPLC results

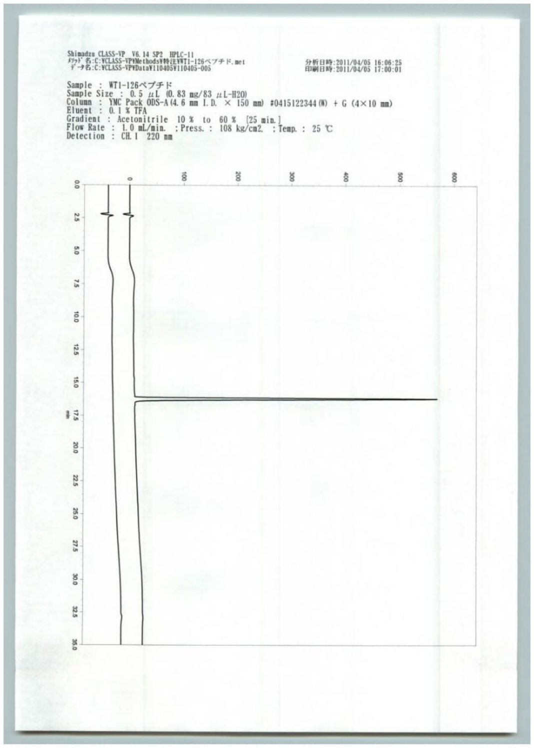

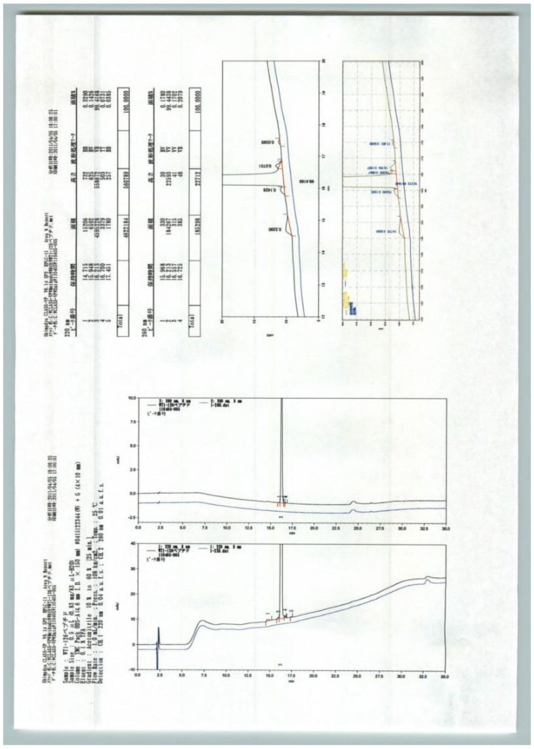

WT1-235

MS

PEPTIDE

株式会社ペプチド研究所
〒567-0085 大阪府茨木市彩都あさぎ７丁目２番９号
Phone:(072)643-4411 Fax:(072)643-4422
PEPTIDE INSTITUTE, INC.
7-2-9 SAITO-ASAGI, IBARAKI-SHI, OSAKA 567-0085, JAPAN
Phone:81-72-643-4411 Fax:81-72-643-4422

品質管理責任者	(stamp)

２０１４年１１月１２日

試験検査報告書

品名	WT1-235 ペプチド
構造	Cys-Tyr-Thr-Trp-Asn-Gln-Met-Asn-Leu
ロット番号	640809
製造日	2014 年 8 月 29 日
容量・数量	1 g×7, 0.5 g×1
検査年月日	2014 年 9 月 1 日～2014 年 10 月 27 日

検 査 項 目	検 査 結 果
1．性状	白色粉末
2．純度試験 *1<br>（規格：90 % 以上）	92% （還元処理［TCEP*7］）<br>80% （未処理）
3．アミノ酸分析*2	構成アミノ酸に由来する 8 種のアミノ酸ピークを認めた<br>Leu を 1 としたときのモル比率<br>Asx (2) 1.979, Thr (1) 0.877, Glx (1) 0.984, Met (1) 0.967, Tyr (1) 0.989, Trp (1) 0.843, Cys (1) 0.834 (Cys 0.422 + 1/2 Cystine 0.412)<br>Leu の回収率 86.67%
4．酢酸*3	5.47%
5．水分*4	3.28%
6．元素分析	実測値：C, 49.07；H, 6.19；N, 13.64 %
7．MS 質量スペクトル	results<br>実測値：m/z=1172.4 （計算値：[M+H]$^+$ exact =1172.479) calculated value
8．残留溶媒*5	ｱｾﾄﾆﾄﾘﾙ：100 ppm 以下； ｼﾞｲｿﾌﾟﾛﾋﾟﾙｴｰﾃﾙ：400 ppm 以下；ｼﾞﾒﾁﾙｽﾙﾎｷｼﾄﾞ：3.47%
9．ｴﾝﾄﾞﾄｷｼﾝ試験*6	0.03 EU/mg 以下
１０．微生物試験	好気性細菌数： 10cfu/0.1g 以下<br>真菌数： 10cfu/0.1g 以下

備考：

HPLC チャート 4 枚添付

*1 日局 16 一般試験法，液体クロマトグラフィーに準じて行う．
カラム：YMC Pack ODS-A, 4.6 mm I.D. × 150 mm
溶離液：0.1% TFA, グラディエント CH_3CN 10% → 60% (25 min)
流速：1.0 mL/min, カラム温度：50℃, 検出：220 nm

*2 加水分解条件：約 10 mg, 4N $MeSO_3H$ (0.2 % Tryptamine 含有) 0.3 mL, 150℃, 2.5 時間

*3, *5 日局 16 一般試験法，ガスクロマトグラフィーに準じて行う．

*4 日局 16 一般試験法，水分測定法（電量滴定法）に準じて行う．

*6 日局 16 一般試験法，ｴﾝﾄﾞﾄｷｼﾝ試験法（ゲル化法）に準じて行う．

*7 トリス (2-カルボキシエチル) ホスフィン塩酸塩

WT1-235

HPLC

Shimadzu CLASS-VP V6.14 SP2 自動HPLC-11 (ｵﾌﾗｲﾝ)
ﾒｿｯﾄﾞ名: C:¥CLASS-VP¥Methods¥特注¥WT1-235.met 分析日時: 2014/10/27 17:24:10
ﾃﾞｰﾀ名: C:¥CLASS-VP¥Data¥141027¥06-試料溶液640809(80%AcOH) 印刷日時: 2014/10/27 19:49:12

Sample : WT1-235ペプチド Lot.640809
Sample Size : 5 μL (2.0 mg/4 mL-80%AcOH)
Column : YMC Pack ODS-A (4.6 mm I.D. × 150 mm) #041593091
Eluent : 0.1% TFA
Gradient : Acetonitrile 10% to 60% [25 min.]
Flow Rate : 1.0 ml/min. , ;Press. : 63 kg/cm2 , ;Temp. : 50 ℃
Detection : CH. 1 220 nm

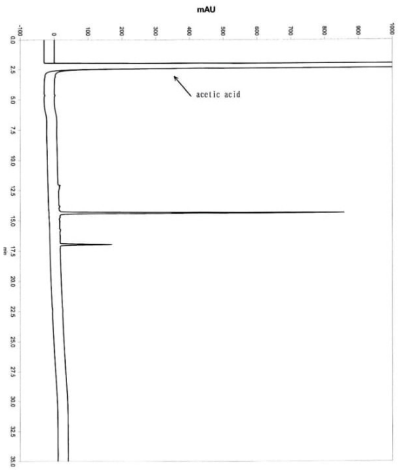

Shimadzu CLASS-VP V6.14 SP2 自動HPLC-11 (ｵﾌﾗｲﾝ)
ﾒｿｯﾄﾞ名: C:¥CLASS-VP¥Methods¥特注¥WT1-235.met 分析日時: 2014/10/27 17:24:10
ﾃﾞｰﾀ名: C:¥CLASS-VP¥Data¥141027¥06-試料溶液640809(80%AcOH) 印刷日時: 2014/10/27 19:49:12

Sample : WT1-235ペプチド Lot.640809
Sample Size : 5 μL (2.0 mg/4 mL-80%AcOH)
Column : YMC Pack ODS-A (4.6 mm I.D. × 150 mm) #041593091
Eluent : 0.1% TFA
Gradient : Acetonitrile 10% to 60% [25 min.]
Flow Rate : 1.0 ml/min. , ;Press. : 63 kg/cm2 , ;Temp. : 50 ℃
Detection : CH. 1 220 nm 0.15 a.u.f.s. : CH. 2 260 nm 0.015 a.u.f.s.

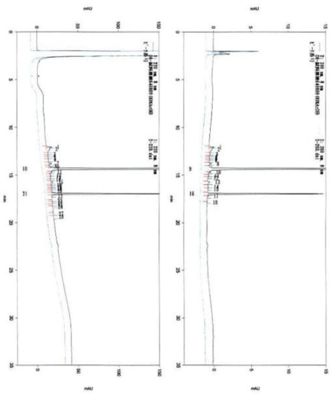

Shimadzu CLASS-VP V6.14 SP2 自動HPLC-11 (ｵﾌﾗｲﾝ)
ﾒｿｯﾄﾞ名: C:¥CLASS-VP¥Methods¥特注¥WT1-235.met 分析日時: 2014/10/27 17:24:10
ﾃﾞｰﾀ名: C:¥CLASS-VP¥Data¥141027¥06-試料溶液640809(80%AcOH) 印刷日時: 2014/10/27 19:49:12

220nm

ﾋﾟｰｸ番号	保持時間	面積	高さ	波形処理ﾏｰｸ	面積%
1	12.076	41609	6709	BV	0.7494
2	12.269	83105	4193	VV	1.4969
3	12.822	20130	1403	VV	0.3626
4	13.003	9551	1020	VV	0.1720
5	13.239	12361	1125	VV	0.2226
6	13.376	13568	870	VV	0.2444
7	13.810	30671	3607	VV	0.5524
8	14.069	7652	920	VV	0.1378
9	14.197	18525	3913	VV	0.3337
10	14.338	4435993	842706	VV	79.8992
11	14.687	22123	2821	VV	0.3985
12	15.045	13952	1514	VV	0.2513
13	15.262	7314	712	VV	0.1317
14	15.488	1560	169	VB	0.0281
15	15.735	27786	2770	BV	0.5005
16	16.064	3910	528	VV	0.0704
17	16.240	9548	1097	VV	0.1720
18	16.373	5412	762	VV	0.0975
19	16.460	6931	888	VV	0.1248
20	16.683	7366	653	VV	0.1327
21	16.947	755382	149225	VV	13.6056
22	17.206	9093	1112	VV	0.1638
23	17.690	1790	322	BV	0.0322
24	17.876	3236	506	VB	0.0583
25	18.187	985	124	BV	0.0177
26	18.342	795	106	VB	0.0143
27	19.072	828	119	BB	0.0149
28	19.360	815	110	BB	0.0147
Total		5551988	1030002		100.0000

260nm

ﾋﾟｰｸ番号	保持時間	面積	高さ	波形処理ﾏｰｸ	面積%
1	12.077	4780	698	BV	0.8387
2	12.288	8773	444	VV	1.5392
3	12.813	3392	251	VV	0.5951
4	13.024	1195	157	VV	0.2096
5	13.230	2058	196	VV	0.3611
6	13.365	1023	151	VV	0.1794
7	13.472	2013	160	VV	0.3532
8	13.811	4170	421	VV	0.7316
9	14.338	458227	87657	VB	80.3964
10	14.687	908	170	TT	0.1593
11	15.065	1057	138	BV	0.1854
12	15.296	312	35	VB	0.0548
13	15.733	2745	290	BV	0.4816
14	16.243	993	100	VV	0.1742
15	16.480	638	70	VB	0.1120
16	16.947	76465	15513	BV	13.4159
17	17.201	825	117	VB	0.1447
18	17.867	387	70	BB	0.0678
Total		569959	106638		100.0000

WT1-235

HPLC

Shimadzu CLASS-VP V6.14 SP2 自動HPLC-11 (ｵﾌﾗｲﾝ)
ﾒｿｯﾄﾞ名: C:¥CLASS-VP¥Methods¥特注¥WT1-235.met 分析日時: 2014/10/03 15:47:42
ﾃﾞｰﾀ名: C:¥CLASS-VP¥Data¥141003¥02-試料溶液640809(TCEP) 印刷日時: 2014/11/12 16:41:44

Sample : WT1-235ペプチド Lot.640809
Sample Size : 10.0 μL (1.009 mg/2 mL-5mM NH4OH with TECP +2mL TFA)
Column : YMC Pack ODS-A (4.6 mm I.D. × 150 mm) #0415164134(W)+G(4×10 mm)
Eluent : 0.1% TFA
Gradient : Acetonitrile 10% to 60% [25 min.]
Flow Rate : 1.0 ml/min. , : Press. : 63 kg/cm2 , : Temp. : 50 ℃
Detection : CH. 1 220 nm

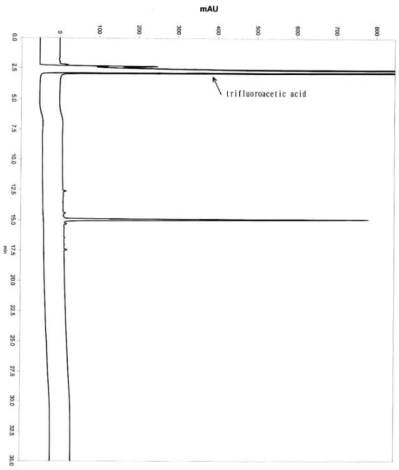

Shimadzu CLASS-VP V6.14 SP2 自動HPLC-11 (ｵﾌﾗｲﾝ)
ﾒｿｯﾄﾞ名: C:¥CLASS-VP¥Methods¥特注¥WT1-235.met 分析日時: 2014/10/03 15:47:42
ﾃﾞｰﾀ名: C:¥CLASS-VP¥Data¥141003¥02-試料溶液640809(TCEP) 印刷日時: 2014/11/12 16:41:44

Sample : WT1-235ペプチド Lot.640809
Sample Size : 10.0 μL (1.009 mg/2 mL-5mM NH4OH with TECP +2mL TFA)
Column : YMC Pack ODS-A (4.6 mm I.D. × 150 mm) #0415164134(W)+G(4×10 mm)
Eluent : 0.1% TFA
Gradient : Acetonitrile 10% to 60% [25 min.]
Flow Rate : 1.0 ml/min. , : Press. : 63 kg/cm2 , : Temp. : 50 ℃
Detection : CH. 1 220 nm 0.15 a.u.f.s. : CH. 2 260 nm 0.015 a.u.f.s.

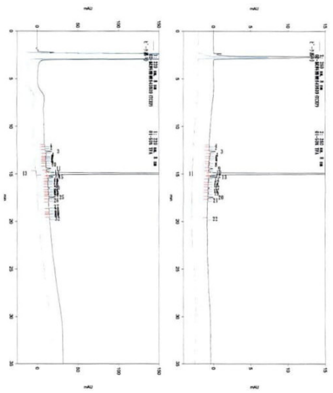

Shimadzu CLASS-VP V6.14 SP2 自動HPLC-11 (ｵﾌﾗｲﾝ)
ﾒｿｯﾄﾞ名: C:¥CLASS-VP¥Methods¥特注¥WT1-235.met 分析日時: 2014/10/03 15:47:42
ﾃﾞｰﾀ名: C:¥CLASS-VP¥Data¥141003¥02-試料溶液640809(TCEP) 印刷日時: 2014/11/12 16:41:44

220nm

ﾋﾟｰｸ番号	保持時間	面積	高さ	波形処理ﾏｰｸ	面積%
1	12.037	1886	291	BV	0.0406
2	12.221	1084	166	VB	0.0234
3	12.620	55876	8579	BV	1.2037
4	12.960	24581	1835	VV	0.5295
5	13.205	4114	692	VV	0.0886
6	13.314	3766	649	VV	0.0811
7	13.419	4130	588	VV	0.0890
8	13.611	801	115	VV	0.0173
9	13.797	4849	679	VV	0.1045
10	14.023	4473	527	VB	0.0964
11	14.427	40240	6038	BV	0.8668
12	14.805	21577	3711	VV	0.4648
13	14.949	4293226	768920	VV	92.4840
14	15.179	15685	3382	VV	0.3379
15	15.316	56216	7807	VV	1.2110
16	15.627	17944	1745	VV	0.3865
17	15.776	4963	898	VV	0.1069
18	15.909	8798	845	VV	0.1895
19	16.181	5116	432	VB	0.1102
20	16.471	11027	2018	BB	0.2375
21	16.757	1253	208	BV	0.0270
22	16.917	889	174	VV	0.0191
23	17.017	6534	905	VV	0.1408
24	17.195	1081	191	VV	0.0233
25	17.444	39174	7292	VB	0.8439
26	17.764	1395	259	BB	0.0301
27	18.448	4429	116	BB	0.0954
28	18.752	1965	236	BB	0.0423
29	19.104	1185	204	BV	0.0255
30	19.230	739	138	VV	0.0159
31	19.349	913	142	VB	0.0197
32	19.695	2220	357	BB	0.0478
Total		4642128	820135		100.0000

260nm

ﾋﾟｰｸ番号	保持時間	面積	高さ	波形処理ﾏｰｸ	面積%
1	12.041	505	82	BV	0.1077
2	12.224	213	29	VB	0.0455
3	12.620	6075	864	BV	1.2970
4	12.958	2821	215	VV	0.6024
5	13.308	1038	120	VV	0.2217
6	13.408	676	125	VB	0.1444
7	13.798	830	158	BV	0.1772
8	14.029	1123	148	VB	0.2397
9	14.428	3560	570	BB	0.7601
10	14.827	1712	347	BV	0.3654
11	14.949	433741	78927	VV	92.6017
12	15.179	1002	281	VV	0.2140
13	15.311	6944	1123	VB	1.4825
14	15.625	1162	118	BV	0.2480
15	15.915	310	44	VB	0.0662
16	16.171	145	22	BB	0.0310
17	16.468	1137	211	BB	0.2428
18	16.760	156	39	BB	0.0333
19	17.015	582	98	BB	0.1242
20	17.444	4161	764	BB	0.8884
21	17.751	205	35	BB	0.0439
22	19.692	295	46	BB	0.0629
Total		468394	84366		100.0000

WT1-332

MS

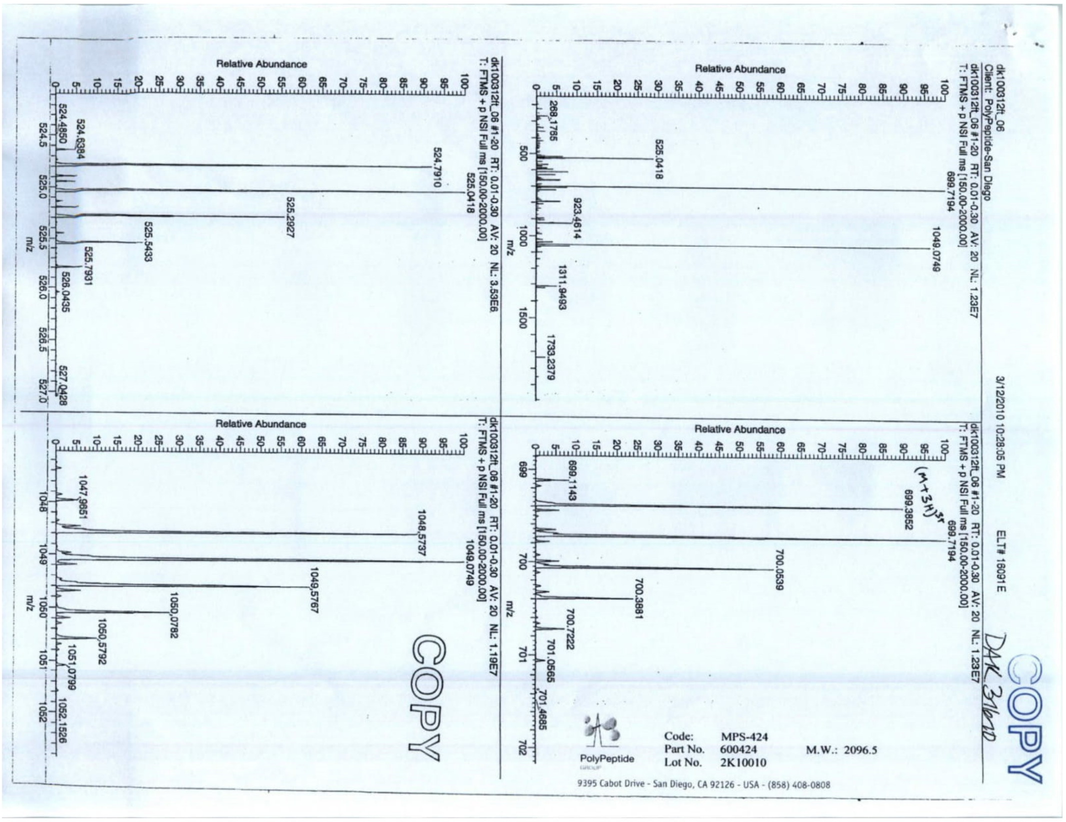
COPY
Code: MPS-424
Part No. 600424
Lot No. 2K10010
M.W.: 2096.5
PolyPeptide
9395 Cabot Drive - San Diego, CA 92126 - USA - (858) 408-0808

WT1-332

HPLC

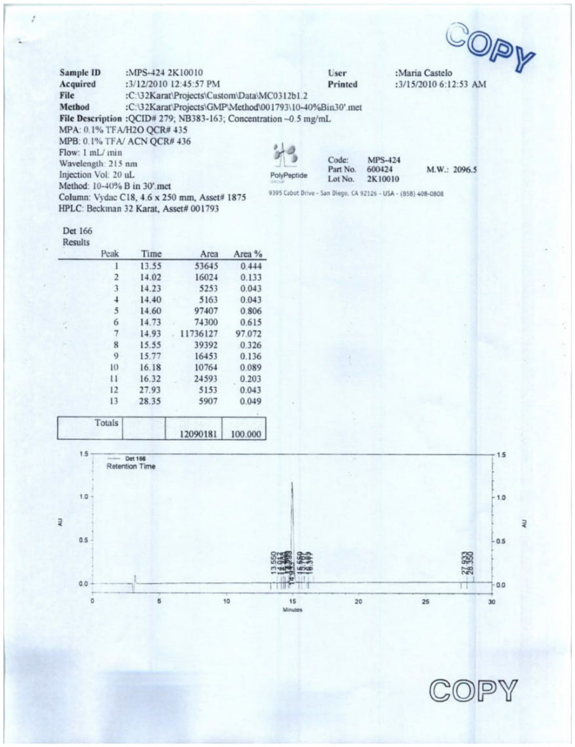

COPY

Sample ID :MPS-424 2K10010 **User** :Maria Castelo
Acquired :3/12/2010 12:45:57 PM **Printed** :3/15/2010 6:12:53 AM
File :C:\32Karat\Projects\Custom\Data\MC0312b1.2
Method :C:\32Karat\Projects\GMP\Method\001793\10-40%Bin30'.met
File Description :QCID# 279; NB383-163; Concentration ~0.5 mg/mL
MPA: 0.1% TFA/H2O QCR# 435
MPB: 0.1% TFA/ ACN QCR# 436
Flow: 1 mL/ min
Wavelength: 215 nm
Injection Vol: 20 uL
Method: 10-40% B in 30'.met
Column: Vydac C18, 4.6 x 250 mm, Asset# 1875
HPLC: Beckman 32 Karat, Asset# 001793

PolyPeptide
Code: MPS-424
Part No. 600424 M.W.: 2096.5
Lot No. 2K10010
9395 Cabot Drive - San Diego, CA 92126 - USA - (858) 408-0808

Det 166
Results

Peak	Time	Area	Area %
1	13.55	53645	0.444
2	14.02	16024	0.133
3	14.23	5253	0.043
4	14.40	5163	0.043
5	14.60	97407	0.806
6	14.73	74300	0.615
7	14.93	11736127	97.072
8	15.55	39392	0.326
9	15.77	16453	0.136
10	16.18	10764	0.089
11	16.32	24593	0.203
12	27.93	5153	0.043
13	28.35	5907	0.049
Totals		12090181	100.000

COPY

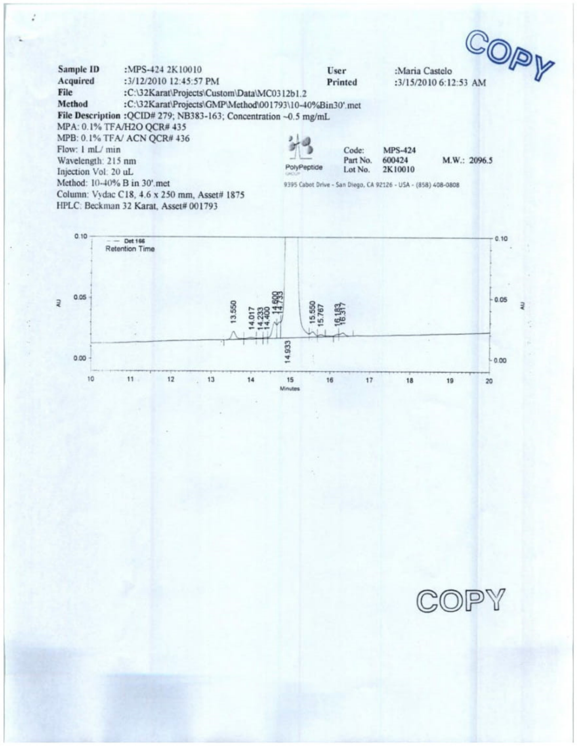

COPY

Sample ID :MPS-424 2K10010 **User** :Maria Castelo
Acquired :3/12/2010 12:45:57 PM **Printed** :3/15/2010 6:12:53 AM
File :C:\32Karat\Projects\Custom\Data\MC0312b1.2
Method :C:\32Karat\Projects\GMP\Method\001793\10-40%Bin30'.met
File Description :QCID# 279; NB383-163; Concentration ~0.5 mg/mL
MPA: 0.1% TFA/H2O QCR# 435
MPB: 0.1% TFA/ ACN QCR# 436
Flow: 1 mL/ min
Wavelength: 215 nm
Injection Vol: 20 uL
Method: 10-40% B in 30'.met
Column: Vydac C18, 4.6 x 250 mm, Asset# 1875
HPLC: Beckman 32 Karat, Asset# 001793

PolyPeptide
Code: MPS-424
Part No. 600424 M.W.: 2096.5
Lot No. 2K10010
9395 Cabot Drive - San Diego, CA 92126 - USA - (858) 408-0808

COPY

Figure S1. WT1 peptide data.
